# The Impact of COVID-19 on Food Consumption and Dietary Quality of Rural Households in China

**DOI:** 10.3390/foods11040510

**Published:** 2022-02-10

**Authors:** Xu Tian, Ying Zhou, Hui Wang

**Affiliations:** 1 College of Economics and Management, Academy of Global Food Economics and Policy, China Agricultural University, Beijing 100083, China; tianxu@cau.edu.cn; 2 College of Economics and Management, Nanjing Agricultural University, Nanjing 210095, China; zhouying_1990@163.com; 3 Department of Maternal and Child Health, School of Public Health, Peking University, Xueyuan Rd 38, Haidian District, Beijing 100191, China

**Keywords:** COVID-19, food consumption, dietary quality, agricultural production

## Abstract

The COVID-19 pandemic disrupted the food supply chain and thus threatened the food security of many people, while the impact of the pandemic on food consumption of people living in rural areas is still unknown. This study filled in the research gaps by employing a three-wave food consumption survey from 2019 to 2021 conducted in rural China. We adopted a random effect model and Poisson regression to quantify the short-run and long-run impacts of COVID-19 on rural households’ food consumption and dietary quality. We found that rural households increased the consumption of vegetables, aquaculture products and legumes in the short-run, and these changes in consumption behavior even lasted 1 year after lockdown was lifted. However, the positive impact was much smaller in households not engaged in agricultural production. In addition, our results showed that COVID-19 decreased dietary diversity but increased dietary quality for households still engaged in food-related agriculture production. Our study indicated that COVID-19 did not threaten the food security status of rural families in China. On the contrary, rural families, particularly those still engaged in agricultural production, increased the consumption of several foods to strengthen their resistance against the virus.

## 1. Introduction

The outbreak and rapid spread of COVID-19 has led to numerous changes in our daily life. To delay and halt the spread of infectious disease, most government have implemented various preventive measures such as lockdown, transportation restriction, and social distancing [1,2]. Those measures effectively contribute to the prevention and control of viral transmission [3,4], but at the cost of a strong negative impact on production and consumption [5]. In particular, the supply chain for many foods such as fruits and vegetables was disrupted [6], which were mainly caused by the lack of a workforce available to harvest and transport fresh food [7,8], closures of various food services such as restaurants [7,9,10], and longer lead time among various distributors due to increased inspections and quarantine measures [11]. Disruption in food supply chains decreased food availability and further led to high price volatility and unaffordability of a healthy and nutritious diet [12,13,14]. Previous studies have found that the pandemic has profoundly reshaped food systems, and changed the way people purchase and consume their food [15,16,17,18,19]. In particular, online shopping became the first choice of many consumers during home confinement [20,21], and the demand for online food groceries increased significantly during the pandemic period [22,23,24]. In addition, consumers’ preference for different foods also changed significantly during and after the pandemic. Several studies revealed that consumers preferred to choose healthy, safe, and better-quality food products during the pandemic [24,25,26], and COVID-19 would contribute to a more sustainable, healthier era of food consumption in the future [27]. However, studies from Chile [28], Greece [29], the UK [30], and several other countries [31] found that people increased the consumption of unhealthy foods such as snacks and pre-packaged “ultra-processed” foods high in fats, sugars and salt during home confinement [32], as those foods had longer shelf life and were easier to access. Consequently, millions of vulnerable people suffered from food insecurity. Previous studies claimed that the share of people who could not afford half of the cost of a nutrient-adequate diet would increase by 7 percentage points during the COVID-19 [12].

Compared with urban residents, some studies argued that people living in rural areas were more vulnerable to COVID-19 as they had limited access to health and medical services, and the rural food market was often poorly developed, particularly in developing countries [1]. By contrast with their counterparts living in urban areas, people living in rural areas can either buy food from market or produce food by themselves. In subsistence farming system, farmers’ food consumption depends on their own production. However, the agricultural sector in developing countries tended to be more specialized and market-oriented in past decades [33], as changing from polyculture to monoculture increased farmers’ productivity and agricultural income [34]. Consequently, decreasing diversity in agricultural production might have a negative impact on the dietary quality of rural residents [35,36,37,38]; meanwhile, farmers’ food consumption became increasingly depended on rural food markets [39]. In addition, agricultural specialization also led to a decreasing demand for agricultural labor, thus many rural residents migrated to urban areas and worked in non-agriculture sectors, and off-farm income became a major income source for many rural families [34]. However, during the pandemic, many rural migrants lost their jobs due to closure of many factories and fell into poverty again [11,40]. Both the disruption in the food supply chain and declining income could negatively affect the food consumption and nutrition status of rural residents. On the other hand, farmers who still cultivated vegetables and raised animals such as chickens, ducks and pigs in vegetable gardens or backyards were less likely to be affected by the pandemic, as their home production could help them to offset the declining food availability from market [41]. Unfortunately, few studies have quantified the effect of COVID-19 on the food consumption and nutrition status of rural people. This study aimed to fill in the research gap by using a 3-year consecutive food survey between 2019 and 2021 conducted in rural China. The research objective was twofold: first, we estimated the short- and long-run impacts of COVID-19 on food consumption and dietary quality of rural residents; second, we tested whether self-production contributed to the resilience of rural residents’ food security by comparing the impact of COVID-19 on food consumption on rural households engaged in agricultural production and those that did not produce any food. 

## 2. Materials and Methods

### 2.1. Study Design and Participants

Data used in this study were collected from a 3-year (2019–2021) consecutive household survey conducted in Jiangsu province, China. The initial survey was conducted by the authors in late April 2019. We selected 417 rural households from 20 villages, in five counties, through a multistage, random cluster survey, which was randomly distributed in Jiangsu province; but only half of them (209) participated in the food consumption survey due to the design of our research program. In late April and early May 2020 we contacted those households again and invited them to participate in an online food consumption survey; 262 out of the 417 households participated in our online survey. One year after the second survey (early May 2021), we contacted these households again and invited them to do a follow-up online survey again; 237 out of the 417 households participated in our third survey. After removing 82 observations with incomplete information or unreliable food consumption value (per capita consumption of one food item was greater than 1 kg), finally we obtained 626 observations which were used in our empirical analysis. The sample size was 198, 220, and 208 in 2019, 2020, and 2021, respectively. Our sample was an unbalanced panel, where 65 households were surveyed three times, 151 households were surveyed twice, and the remaining 129 only participated in one round of the survey.

### 2.2. Food Consumption Data and Dietary Quality

Food consumption for the whole family was recorded using 24-h recall method. We recorded the quantity, location and time of consumed poultry, beef and mutton, pork, aquaculture products, eggs, fruits, vegetables, cereals, tubers, dairy and dairy products, legumes, and nuts and seeds in each surveyed family in the day (24 h) before our survey. Only food eaten at home was recorded. Meanwhile, we also recorded the number of meals eaten at home and away from home for all family members, and the total frequency of meals ate at home were used to estimate the person-day for the whole family. We then calculated food consumption per capita per day by dividing the family food consumption by person-day. We further combined all food items into eight food groups according to the China Food Pagoda (CFP) 2016, namely meat (poultry, beef and mutton, pork), aquaculture products, eggs, fruits, vegetables, grains (cereals and tubers), dairy and dairy products, and legumes (including nuts and seeds). 

Dietary quality was measured by two indicators: the dietary diversity and the Chinese Food Pagoda Score (CFPS). Dietary diversity was the number of food groups consumed by each household. CFPS was estimated by the deviation between the real dietary pattern and the recommended dietary pattern in the CFP 2016. The CFP 2016 demonstrated the main principles of the Chinese Dietary Guidelines 2016 (CDG 2016) in a figure, and transformed the principle into recommended daily consumption quantity (lower and upper bounds) for healthy adults for 10 food groups. We followed the methodology proposed by Huang and Tian [33], and took the reference for 2200 kcal (mean daily calorie intake per capita in China based on previous studies) as standard to calculate the scores for each food item (detailed assignment methods of various foods in the CFP 2016 are shown in Table 1). Finally, we summed up the scores of eight food groups. Bear in mind that we did not collect data on edible oil and salt (edible oil and salt were not included in our survey due to technical difficulty to record daily consumption correctly), so that CFPS in our study ranged from 0 to 8, and a greater CFPS indicated a more balanced diet which converged to the CFP 2016. 

### 2.3. COVID-19 Data

The Chinese Center for Disease Control and Prevention (http://2019ncov.chinacdc.cn/2019-nCoV/, accessed on 9 January 2022). updated city-level COVID-19 data in each day since early 2020. At the time of our second-round survey (April and May 2020), the cumulative COVID-19 cases in our surveyed cities ranged from 36 to 101. Those number did not change in the following year due to effective preventive measures. In our study, we adopted a binary variable to capture the impact of COVID-19, which was set to be 0 in 2019 and 1 in 2020 and 2021. This was so that the coefficient of COVID-19 dummy could be explained as the changes in food consumption and dietary quality between 2019 and 2020 (2021).

### 2.4. Other Co-Variants

We controlled characteristics of household (household size, share of old people in the family, share of children in the family, household income), and characteristics of household head (age, gender, marital status, education) as co-variants in our regression. The diversity of agricultural production (the number of food groups ever produced by household) was also included as covariant to control the potential linkage between own production and food consumption. In addition, village dummies were controlled as well to remove the heterogeneity in medical service, human mobility, and preventive polices across different regions. 

### 2.5. Empirical Strategy

As our sample is not a strongly balanced panel data, we employed a multivariable regression model to estimate the impact of COVID-19 on food consumption, which could control other factors (aforementioned co-variants) that may also affect household food consumption behavior. We adopted both a random-effect (RE) model and pooled ordinal least regression (OLS) to estimate the model. In the main text, we only report results estimated using RE, and results estimated using OLS are presented in the Supplementary File.

The impact of COVID-19 on dietary diversity was estimated using a Poisson regression, which was better to model nonnegative integer data (number of food items). The impact of COVID-19 on CFPS was estimated using RE and OLS. 

We first estimated the impact of COVID-19 on rural households’ food consumption and dietary quality using data in 2019 and 2020. In April 2020 all lockdown cities in China had reopened but people were still concerned about the pandemic and most of them did not return to work. This was so that the changes in food consumption from 2019 to 2020 could be treated as a short-run impact of COVID-19. One year after lockdowns were lifted (May 2021), about half of Chinese citizens had been vaccinated for the COVID-19. The economy recovered, production rebounded and life got back to normal. Therefore, we treated the changes in food consumption from 2019 to 2021 as the long-run impact of COVID-19. 

Furthermore, food consumption of many rural households still depended on own production [41]. We thus further divided our sample into two subsamples based on whether household still participated in any agriculture production (food-related agricultural activity), and re-estimated all regressions for two sub-samples again. We named rural households who were still engaged in agricultural production as farmers, and named rural households who did not produce any foods as non-farmers. 

All statistical analyses were conducted with Stata 14.0 (Stata Corp., College Station, TX, USA). Statistical significance was defined at 5%. 

## 3. Results

### 3.1. Food Consumption and Dietary Quality in Three Years

Table 2 summarizes the mean and standard deviation of all variables in each year. We found statistically significant difference in five out of eight food categories (all *p* < 0.05). In particular, consumers increased the consumption of vegetables, fruits, meat, aquaculture products, and legumes in 2020 and 2021. But their dietary diversity decreased significantly after 2019, while a slight increase in CFPS was observed in 2020 but dropped 1 year later (all *p* < 0.05). 

**Figure 1:**
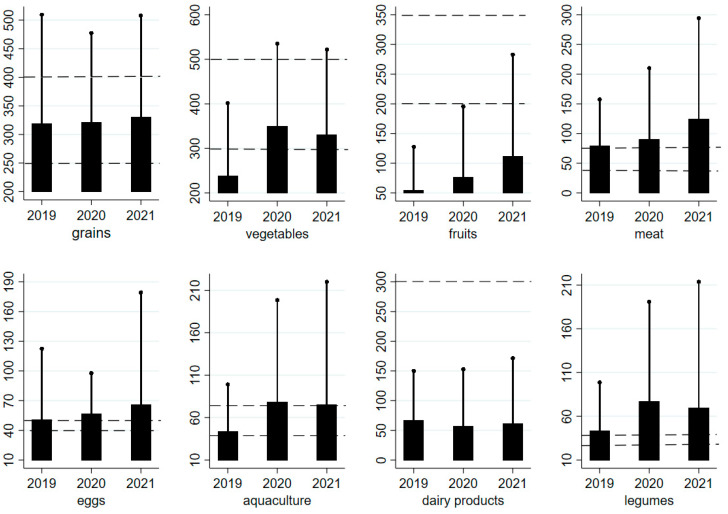
**Food consumption in three years**. Meat includes livestock and poultry, grains includes cereals and tubers, dairy products includes milk and dairy products, legumes includes legumes, nuts and seeds. The vertical axis shows daily per capita food consumption measured in grams. The horizontal dotted lines show the upper and lower recommended intakes for each food category. The upper and lower limits are 40/75, 40/75, 40/50, 200/350, 300/500, 250/400, 300, 25/35 (g/day) for meat, aquatic products, eggs, fruits, vegetables, grains, legumes, respectively. In the Chinese Dietary Guidelines, only 300 g/day (lower limit) is set for milk and dairy products. The bar chart shows the average daily per capita food consumption by all rural households. The short black solid line at the top of the bar chart represents the mean + standard deviation.

A more intuitive demonstration of the changes in food consumption is presented in Figure 1. The consumption of all foods, except for dairy products, increased after the pandemic, and a significant increase was observed in vegetables, fruits, aquaculture products, and legumes.

**Figure 2:**
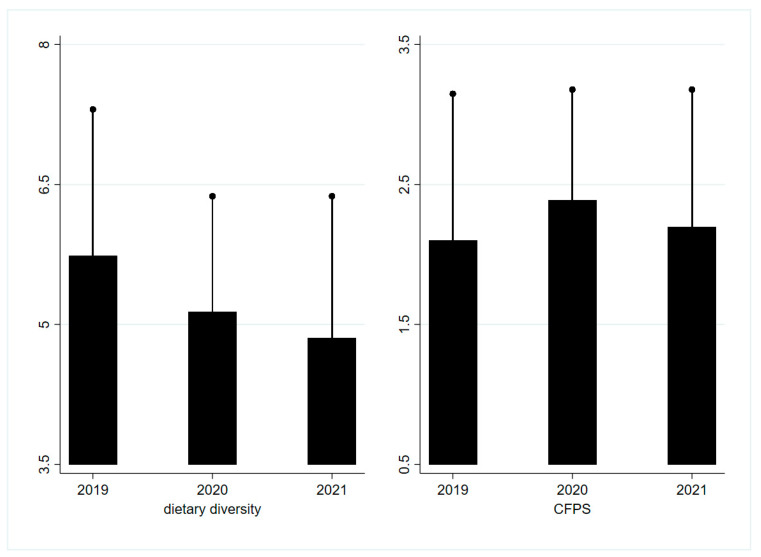
**Dietary quality over 3 years**. Dietary diversity is measured by the number of 12 food items consumed by household in the survey data. CFPS refers to China Food Pagoda Score. The bar chart and short black solid line at the top of the bar represent the mean and mean + standard deviation.

Figure 2 presents two dietary quality indicators in three years. Data showed that dietary diversity decreased year by year, while CFPS increased after the pandemic. 

### 3.2. Impact of COVID-19 on Food Consumption

We did not find any evidence that COVID-19 had negative impact on rural households’ consumption of any food category (Table 3). On the contrary, the consumption of seven out of eight food categories increased in 2020, and three of them (vegetables, aquaculture products, and legumes) increased significantly. In particular, daily per capita consumption of vegetables, aquaculture products, and legumes increased by 119.61, 29.85 and 29.36 g respectively (all *p* < 0.05). The only negative impact was observed in dairy products, but the declining consumption was statistically insignificant. 

One year after lockdowns were lifted, the positive impact of COVID-19 on households’ food consumption persisted and became even stronger. We found that five out of eight food categories experienced significant increase in 2021. The daily per capita consumption of vegetables, fruits, meat, aquaculture products, and legumes increased by 92.92, 37.24, 27.88, 29.47 and 24.48 g, respectively (all *p* < 0.05).

The results for two sub-samples are presented in Table 4. We found that COVID-19 significantly increased the consumption of several food categories for farmers, but the impact was much smaller for non-farmers. In 2020, agricultural households’ consumption of vegetables, aquaculture products, and legumes increased by 137.28, 28.08 and 25.35 g, respectively (all *p* < 0.05). By contrast, non-farmers’ consumption of aquaculture products and legumes increased by 53.38 and 58.90 g, but their consumption of meat decreased by 106.42 g (*p* < 0.05). These impacts lasted even after 1 year, but the negative impact on non-farmers became statistically insignificant. 

### 3.3. Impact of COVID-19 on Dietary Quality

We found that COVID-19 significantly reduced rural households’ dietary diversity by 0.64 and 0.75 in the short-run and long-run, respectively, but the negative impact was only statistically significant for farmers (all *p* < 0.05, Table 5). However, COVID-19 significantly increased CFPS by 0.24 in the short-run, but the impact was only significant for farmers in the short-run (all *p* < 0.05, Table 5).

### 3.4. Robustness Check

We conducted all regressions again using the OLS method. Results were presented in Supplementary File Tables S9–S17. In general, all results estimated from OLS were consistent with that from RE, indicating that our main findings are robust.

## 4. Discussion

This study quantified the impact of COVID-19 on food consumption and dietary quality of rural households using both pre- and post-pandemic food consumption surveys conducted in Jiangsu, China. We found that rural families increased their consumption of vegetables, aquaculture products, and legumes, and these changes in food consumption persisted 1 year after lockdowns were lifted. In addition, we found that dietary diversity decreased slightly both in the short-run and long-run, but CFPS increased in the post pandemic period for households who were still engaged in at least one food-related agricultural production.

Previous studies claimed that food production declined and disruption in food supply chain would create food price spikes and local food shortages [14], and those shocks would lead to greater unaffordability of healthy and nutritious diets in low- and middle-income countries [12]. However, these studies mainly estimated the impact of COVID-19 on diet and nutrition in the early stages of the pandemic, when food supply chains were totally disrupted and fresh foods were facing great shortages [11,13], thus many vulnerable people were very likely to be affected by the pandemic. In addition, previous studies mainly focused on urban area or countries where agricultural production was highly specialized [7,42], so that citizens in those areas depended on food markets to purchase food. During the pandemic, almost all countries implemented various preventive measures to delay the transmission of the disease [3,43]. Those policies such as travel restrictions, social distancing, inspections and quarantine measures, decreased the availability of the workforce to harvest and transport fresh food from farm to cities [7,8,42], and increased lead time among distributors [11]. In addition, many formal and informal food services such as restaurants and supermarkets in urban areas were closed or under strong restrictions [11]. All of those aforementioned factors created several bottlenecks from farm to fork [11]. In addition, a large proportion of households have been exposed to job losses or reduced incomes during the pandemic. The declining purchasing power further worsened families’ access to food, particularly among the vulnerable [11].

On the contrary, we observed significant positive impact of COVID-19 on food consumption and dietary quality. The difference could be attributable to the following reasons. First, our second round of survey was conducted at the end of April 2020, when all cities in China (including Wuhan) had reopened and the food supply chain was re-built, so that no one in our sample had serious problem of accessing to food. Second, our study focused in rural China where many foods, particularly fresh foods, were produced, so that people were less likely to be affected by food shortages. Moreover, most rural families cultivated vegetables and fruits in vegetable gardens around their houses for self-consumption. Some rural households even raised several chicken, ducks or pigs in their garden using swill feeding produced from kitchen. The quasi-subsistent farming system diversified their food supply channels, and contributed to resilience of food consumption during the pandemic. Our data showed that about 1/3 foods consumed in rural families in China were self-produced, and own production played an important role in food consumption in rural China. Third, China had built a very efficient food supply chain in past decades. As early as the late 1980s, the ministry of agriculture in China implemented the “Shopping basket program”, which aimed to support the production, logistics, storage, and processing of main food items including meat, eggs, dairy products, aquaculture products, fruits and vegetables. After three decades of rapid development, China had built a diverse and resilient food supply system with about 200 million farms at various scales [44]. Many studies claimed that building redundancy and diversity in food system was essential for resilience of the food system in the COVID-19 pandemic [1,7,8]. Even during the complete lockdown period, the Chinese government still delivered sufficient foods from various sources to families under home confinement.

An alternative explanation is that rural families had limited access to medical services, thus increasing the consumption of healthy foods was a cost-effective strategy to cope with the increasing uncertainty in COVID-19-related health risk [41,45]. In addition, people tended to eat more foods during quarantine due to mood-driven eating such as anxiety and boredom [46]. Previous studies found evidence that people increased the consumption of unhealthy foods such as snacks, foods high in salt, sugar, and fats during home confinements in several countries [28,29,30,31,32], and the overeating combined with declining physical activity could lead to weight gains [28,47]. Luckily our study found that rural families increased the consumption of vegetables, aquaculture products and legumes. The first two were insufficiently consumed before the pandemic (see Figure 1), thus higher consumption contributed to better dietary quality. However, legumes and meat were already over-consumed in 2019, thus growing consumption in 2020 and 2021 led to unbalanced diets, which might increase overweight/obesity risk for rural people. The overconsumption of meat was a big concern in China given the fact that more than 50% of adults were affected by overweight and obesity in China, and fat accounted for more than 30% of total energy intake [48].

Finally, we also found that COVID-19 had a stronger positive impact on rural households who still engaged in food-related agriculture production (farmers). Consequently, farmers’ dietary diversity declined slightly, but their dietary quality increased after the COVID-19. Our findings indicated that own production still contributed to the resilience of the food system in rural areas. Previous studies also found that agricultural production diversity was positively associated with dietary diversity of farmers in several developing countries [35,36,37,49], but this impact would be weakened and tended to be insignificant if farmers can easily access food market [33,35,50]. During the COVID-19, the disruption in food supply chain reduced food availability in markets, and thus had negative impact on the accessibility of food. But farmers who still cultivated vegetables/fruits and raised animals/fish in backyards or ponds were less likely to be affected, and spent more time on their vegetable gardens and ponds to increase harvest. The increasing time allocated to agricultural production could offset the negative impact of declining market accessibility, and further contributed to increased production and consumption of vegetables, fruits, aquaculture products and legumes. The changing dietary pattern, particularly the increasing consumption of vegetables and fruits which were seriously under-consumed before the COVID-19, improved the dietary quality of farmers.

Several limitations should be mentioned in this study: First, our study was conducted in Jiangsu province, which is one of the richest provinces in China and was famous for its aquaculture and rice production (accounted for 7.5% and 9.3% of national production [51]). Rural areas in Jiangsu had a well-developed food supply system and thus people were less likely to be affected by food shortages. However, in rural areas in southwest and northwest China, infrastructure is poorly developed and households live far away from each other and food markets, and rural families were more likely to suffer from food shortages during the pandemic. Second, data in 2020 and 2021 were collected via an online telephone survey due to restrictions on field survey. Thus they might have bigger measurement errors than data in 2019, as people tended to be less patient in online surveys. In addition, there might be a selection bias if household participated in the online survey differ significantly from those refused to participate. Nevertheless, we still contributed to current literature in the following points. First, to our knowledge, this is the first study that quantified the impact on COVID-19 on food consumption and dietary quality of rural families in developing countries. Second, our study provides evidence that self-production contributes to resilience of food consumption in rural area in response to unexpected shocks. Combined with the finding from Huang and Tian [33] who found that the diet of families engaged in diverse agricultural production was not affected by food accessibility in local markets, we claimed that self-production can still contribute to farmers’ food security status in light of the pandemic. Thus a diverse food supply chain can contribute to the resilience of the food supply system and improve dietary quality in rural areas.

## 5. Conclusions

This study found that rural residents’ dietary diversity decreased slightly after the COVID-19 pandemic, but they increased the consumption of vegetables, aquaculture products, and legumes to strengthen their resistance against the virus. These impacts lasted 1 year after lockdown was lifted, and was stronger for households that still engaged in food-related agricultural production (farmers). Consequently, the dietary quality of farmers increased after the COVID-19.

## Figures and Tables

**Table 1:** Measurement of Chinese Food Pagoda Score (CFPS).

Food Group	Consumption	Dietary Guidelines
Grains, potatoes and beans (g)		250–400
Score as “1”	250–300	
Score as “0.5”	125–250	
Score as “0.5”	300–450	
Score as “0”	≥450 or ≤125	
Vegetables (g)		300–500
Score as “1”	≥450	
Score as “0.5”	225–450	
Score as “0”	≤225	
Fruit (g)		200–350
Score as “1”	≥300	
Score as “0.5”	150–300	
Score as “0”	≤150	
Meat and poultry (g)		40–75
Score as “1”	50–100	
Score as “0.5”	25–50	
Score as “0.5”	100–150	
Score as “0”	≥150 or ≤25	
Eggs (g)		40–50
Score as “1”	40–50	
Score as “0.5”	20–40	
Score as “0.5”	50–75	
Score as “0”	≥75 or ≤20	
Aquatic products (g)		40–75
Score as “1”	≥75	
Score as “0.5”	38–75	
Score as “0”	≤38	
Milk and its products (g)		300
Score as “1”	≥300	
Score as “0.5”	150–300	
Score as “0”	≤150	
Legumes and nuts(g)		25–35
Score as “1”	25–35	
Score as “0.5”	13–25	
Score as “0.5”	35–53	
Score as “0”	≥53 or ≤13	

Note: Each food group gets score ‘1′ if the real consumption locates within the recommended consumption interval. If the real consumption is 50% higher than the upper bound or 50% lower than the lower bound, the score will be set as ‘0.5′. If the deviation between real consumption and recommendation is too large, the score will be set as ‘0′. Furthermore, in order to encourage a healthy diet, the score is set as ‘1′ if the real consumption of fruit, vegetable and aquaculture products is greater than a given value. Finally, the score of eight food groups is summed up to calculate the CFPS for each individual. Therefore, a greater CFPS indicates a more balanced diet which adheres to the CFP 2016.

**Table 2:** Summary of all variables in three waves.

Variable	2019 (*n* = 198)		2020 (*n* = 220)		2021 (*n* = 208)		Mean Equality Test
	Mean	Std. Dev.	Mean	Std. Dev.	Mean	Std. Dev.	
Food Consumption							
Grains	318.97	190.37	320.70	156.54	329.89	178.03	0.45
Vegetables	238.00	163.82	348.94	186.14	330.15	191.67	48.68 *
Fruits	54.72	72.86	76.85	118.85	111.92	170.89	21.51 *
Meat	79.33	78.29	89.93	120.17	124.33	169.80	12.00 *
Eggs	50.79	71.58	56.44	41.41	65.98	113.29	2.69
Aquaculture	43.96	55.04	78.27	120.15	75.20	144.70	19.97 *
Dairy products	66.61	83.27	56.92	96.07	61.58	109.98	1.22
Legumes	43.09	55.58	76.83	114.04	69.61	144.05	18.61 *
Dietary Quality							
Dietary diversity	5.73	1.57	5.14	1.44	4.86	1.52	33.73 *
CFPS	2.10	1.05	2.37	0.99	2.19	0.98	8.68 *
Household Characteristics							
ln(income)	1.71	0.88	1.42	0.86	1.83	0.87	24.65 *
Household size	4.16	1.85	3.83	1.74	3.52	1.59	13.96 *
old_share	0.38	0.34	0.42	0.39	0.55	0.37	23.40 *
children_share	0.12	0.14	0.10	0.13	0.12	0.15	3.23
Production diversity	2.59	1.53	1.26	1.64	2.17	1.79	75.91 *
Household Head Characteristics							
Age	63.98	10.11	64.59	10.02	63.77	9.33	0.81
Gender(male)	0.92	0.27	0.95	0.22	0.93	0.26	1.85
Marital status	0.91	0.27	0.94	0.24	0.92	0.27	1.09
Education	7.93	3.52	8.14	3.31	8.38	3.24	1.86

Note: Dietary diversity counts the number of food groups consumed daily, which ranges from 0 to 12. CFPS refers to the Chinese Food Pagoda Score. ln(income) refers to the household income. Old_share and children_share measure the share of old people (60+) and children (<18) in each family. Production diversity counts the number of food groups ever produced by household. Age, gender and marital status refer to household head’s age, gender and marital status (single or married). Education captures the year of formal education attended by household head. * refers to statistically significant at 5%.

**Table 3:** Impact of COVID-19 on the consumption of eight food categories.

Food Category	Short-Run	Long-Run
grains	7.71	3.60
vegetables	119.61 *	92.92 *
fruits	18.85	37.24 *
meat	12.76	27.88 *
eggs	9.49	10.55
aquaculture	29.85 *	29.47 *
dairy products	−9.52	−6.33
legumes	29.36 *	24.48 *

Note: Results were estimated using Radom-Effect model. Short-run impact was estimated using data collected in 2019 and 2020, and long-run impact was tested using data collected from 2019 to 2021. Characteristics of household (income, household size, share of children and old people in the household, diversity of agricultural production) and household head (age, gender, marital status, education), and village dummies had been adjusted in the regression. * refers to statistically significant at 5%. Complete regression results were presented in Tables S1 and S2.

**Table 4:** Impact of COVID-19 on the consumption of eight food categories for farmers and non-farmers.

Food Category	Farmers		Non-Farmers	
	Short-Run	Long-Run	Short-Run	Long-Run
grains	6.99	−1.83	−14.11	8.03
vegetables	137.28 *	100.59 *	−17.01	−24.61
fruits	23.41	36.90 *	5.26	6.54
meat	28.23	27.39 *	−106.42 *	−63.98
eggs	8.07	7.87	15.75	44.39
aquaculture	28.08 *	26.46 *	53.38 *	73.88 *
dairy products	−7.88	−9.21	−30.61	−30.59
legumes	25.35 *	18.94 *	58.90 *	56.02 *

Note: Results were estimated using Radom-Effect model. Short-run impact was estimated using data collected in 2019 and 2020, and long-run impact was tested using data collected from 2019 to 2021. Characteristics of household (income, household size, share of children and old people in the household, diversity of agricultural production) and household head (age, gender, marital status, education), and village dummies had been adjusted in the regression. Farmers referred to rural households who were still engaged in agricultural production, and non-farmers was the sample only includes rural households who did not produce any foods. * refers to statistically significant at 5%. Complete regression results were presented in Tables S3–S6.

**Table 5:** Impact of COVID-19 on dietary diversity and CFPS.

Quality	Total Sample		Farmers		Non-Farmers	
	Short-Run	Long-Run	Short-Run	Long-Run	Short-Run	Long-Run
Dietary diversity	−0.64 *	−0.75 *	−0.60 *	−0.78 *	−0.58	−0.66
CFPS	0.24 *	0.17	0.30 *	0.17	0.38	0.29

Note: Results were estimated using Radom-Effect model. Short-run impact was estimated using data collected in 2019 and 2020, and long-run impact was tested using data collected from 2019 to 2021. Dietary diversity was the number of food items consumed by each household. CFPS was the Chinese Food Pagoda Score. Characteristics of household (income, household size, share of children and old people in the household, diversity of agricultural production) and household head (age, gender, marital status, education), and village dummies had been adjusted in the regression. Farmers referred to rural households who were still engaged in agricultural production, and non-farmers was the sample only includes rural households who did not produce any foods. * refers to statistically significant at 5%. Complete regression results were presented in Tables S7 and S8.

## Data Availability

Data is acceptable upon request.

## Supplementary Materials

The following supporting information can be downloaded at: https://www.mdpi.com/article/10.3390/foods11040510/s1, Table S1: Short-run impact of COVID-19 on the consumption of eight food categories; Table S2: Long-run impact of COVID-19 on the consumption of eight food categories; Table S3: Short-run impact of COVID-19 on the consumption of eight food categories for farmers; Table S4: Long-run impact of COVID-19 on the consumption of eight food categories for farmers; Table S5: Short-run impact of COVID-19 on the consumption of eight food categories for non-farmers; Table S6: Long-run impact of COVID-19 on the consumption of eight food categories for non-farmers; Table S7: Impact of COVID-19 on dietary diversity and CFPS; Table S8: Impact of COVID-19 on dietary diversity and CFPS for farmers and non-farmers; Table S9: Short-run impact of COVID-19 on the consumption of eight food categories; Table S10: Long-run impact of COVID-19 on the consumption of eight food categories; Table S11: Short-run impact of COVID-19 on the consumption of eight food categories for farmers; Table S12: Long-run impact of COVID-19 on the consumption of eight food categories for farmers; Table S13: Short-run impact of COVID-19 on the consumption of eight food categories for non-farmers; Table S14: Long-run impact of COVID-19 on the consumption of eight food categories for non-farmers; Table S15: Impact of COVID-19 on dietary diversity and CFPS; Table S16: Short-run impact of COVID-19 on dietary diversity and CFPS for farmers and non-farmers; Table S17: Long-run impact of COVID-19 on dietary diversity and CFPS for farmers and non-farmers.
