# Supplementary material for: The Impact of COVID-19 on Food Consumption and Dietary Quality of Rural Households in China
Source: Foods. 2022 Feb 10;11(4):510. doi: 10.3390/foods11040510 (PMC8870752; doi:10.3390/foods11040510)
Supplement: Supplementary file 1 [file foods-11-00510-s001.zip › foods-1571616-supplementary.pdf]

Supplementary file:

Table S1 Short-run impact of COVID-19 on the consumption of 8 food categories

Variable	grains	vegetables	fruits	meat	eggs	aquacultu re	dairy products	legumes
COVID-19	7.71	119.61*	18.85	12.76	9.49	29.85*	-9.52	29.36*
ln(income)	-2.06	11.48	5.65	12.25	2.02	-3.21	3.84	1.76
household size	-17.69*	-28.76*	-5.81	-6.32	-6.98*	-1.37	-7.72	-5.19
old_share	-51.23	15.67	-18.82	-28.16	-31.33*	-22.50	-19.85	35.19
children_share	-118.60	-31.29	-41.03	-10.49	-36.23	-124.66*	76.88	-26.06
production diversity	5.88	5.18	-2.09	-0.48	3.69	-1.06	0.68	-1.74
Age	0.35	-1.30	1.08*	1.03	0.13	-0.31	1.37*	-0.59
Gender(male)	34.21	-65.32	-42.58	1.10	10.56	-5.17	-45.97	24.66
Marital status	-30.77	53.63	-9.51	-13.63	-11.87	7.82	14.55	-55.27*
Education	-7.83*	-8.07*	2.70	0.33	-0.18	0.68	3.37*	-0.21
Village control	Yes	Yes	Yes	Yes	Yes	Yes	Yes	Yes
Constant	499.69*	573.82*	48.75	48.61	121.04*	98.87	-10.88	91.20*
Observations	418	418	418	418	418	418	418	418
Number of hhid	315	315	315	315	315	315	315	315
Mean of Y	319.90	296.40	66.37	84.91	53.76	62.02	61.51	60.85

Note: Results were estimated using Radom-Effect model and using data collected in 2019 and 2020. Characteristics of household (income, household size, share of children and old people in the household, diversity of agricultural production) and household head (age, gender, marital status, education), and village dummies had been adjusted in the regression. Number of hhid is the number of households net of duplicated observations. * refers to statistically significant at 5%.

Table S2 Long-run impact of COVID-19 on the consumption of 8 food categories

Variable	grains	vegetables	fruits	meat	eggs	aquacultu re	dairy products	legumes
COVID-19	3.60	92.92*	37.24*	27.88*	10.55	29.47*	-6.33	24.48*
ln(income)	8.63	11.57	23.97*	19.19*	15.15*	-0.19	8.89	7.43
household size	-22.43*	-27.59*	-3.50	-12.77*	-4.84	-0.67	-7.85	-3.63
old_share	-0.66	15.39	22.00	-17.60	-27.17	-7.72	-1.55	23.47
children_share	53.55	-49.54	-2.43	61.38	-64.33	-107.51*	78.20	-46.71
production diversity	2.22	0.22	-1.00	1.77	1.62	-0.93	4.16	-2.95
Age	-0.01	-0.42	0.41	0.45	0.11	0.01	0.15	-0.33
Gender(male)	23.58	-20.20	-35.87	-12.60	-0.56	13.38	-7.05	54.23*
Marital status	-1.30	16.33	-39.90	10.61	-4.09	-22.46	-14.43	-99.85*
Education	-7.67*	-6.69*	5.13*	1.37	-0.29	3.20	3.31*	1.81
Village control	Yes	Yes	Yes	Yes	Yes	Yes	Yes	Yes
Constant	487.05*	493.54*	45.31	50.22	101.49*	30.18	25.92	79.70
Observations	626	626	626	626	626	626	626	626
Number of hhid	345	345	345	345	345	345	345	345
Mean of Y	323.2	307.6	81.50	98.01	57.82	66.40	61.53	63.76

Note: Results were estimated using Radom-Effect model and using data collected from 2019 to 2021. Characteristics of household (income, household size, share of children and old people in the household, diversity of agricultural production) and household head (age, gender, marital status, education), and village dummies had been adjusted in the regression. Number of hhid is the number of households net of duplicated observations. * refers to statistically significant at 5%.

Table S3 Short-run impact of COVID-19 on the consumption of 8 food categories for farmers

Variable	grains	vegetables	fruits	meat	eggs	aquaculture	dairy products	legumes
COVID-19	6.99	137.28*	23.41	28.23	8.07	28.08*	-7.88	25.35*
ln(income)	4.81	14.50	4.66	12.72	4.97	0.39	0.21	4.58
household size	-20.39*	-30.37*	-5.47	-10.58*	-10.08*	2.04	-8.66	-5.42
old_share	-75.91	-20.34	-25.71	-13.58	-44.18	6.76	8.32	57.53*
children_share	-178.77	-62.69	-50.33	-4.84	-42.06	-103.41	108.75	16.80
production diversity	9.51	3.43	-3.93	-3.99	4.18	1.34	0.40	0.40
Age	0.67	-0.78	0.76	0.15	-0.17	-0.90	1.12	-1.15*
Gender(male)	33.14	-68.66	2.77	33.89	15.75	-1.98	-41.12	61.87*
Marital status	-46.05	61.65	5.33	-46.18	-13.92	-19.94	33.38	-69.27*
Education	-8.75*	-10.25*	1.93	-0.60	-0.38	0.46	2.56	-0.90
Village control	Yes	Yes	Yes	Yes	Yes	Yes	Yes	Yes
Constant	537.39*	631.97*	55.09	157.61*	184.02*	108.71	-10.63	108.39*
Observations	289	289	289	289	289	289	289	289
Number of hhid	193	193	193	193	193	193	193	193
Mean of Y	319.90	296.40	66.37	84.91	53.76	62.02	61.51	60.85

Note: Results were estimated using Radom-Effect model and using data collected in 2019 and 2020. Characteristics of household (income, household size, share of children and old people in the household, diversity of agricultural production) and household head (age, gender, marital status, education), and village dummies had been adjusted in the regression. Farmers referred to rural households who were still engaged in agricultural production. Number of hhid is the number of households net of duplicated observations. * refers to statistically significant at 5%.

Table S4 Long-run impact of COVID-19 on the consumption of 8 food categories for farmers

Variable	grains	vegetables	fruits	meat	eggs	aquaculture	dairy products	legumes
COVID-19	-1.83	100.59*	36.90*	27.39*	7.87	26.46*	-9.21	18.94*
ln(income)	14.01	13.83	26.30*	15.92*	17.99*	2.58	9.46	10.97*
household size	-26.08*	-27.05*	1.74	-9.38*	-5.75	3.23	-8.58	-2.09
old_share	-4.87	4.30	34.62	15.90	-23.21	28.84	22.87	48.48*
children_share	59.62	-57.02	-47.45	40.55	-88.49	-82.19	97.45	-18.18
production diversity	5.11	3.03	-4.63	-0.09	1.10	-0.29	6.82*	-3.64
Age	0.26	-0.42	-0.25	-0.55	-0.03	-0.75	-0.32	-0.97
Gender(male)	23.16	-22.34	-7.07	5.51	-5.01	25.85	1.58	75.50*
Marital status	0.86	-0.41	-35.49	-11.35	-4.01	-57.02*	-23.89	-125.29*
Education	-9.82*	-7.53*	5.31*	-0.03	-0.23	3.43	3.35*	1.78
Village control	Yes	Yes	Yes	Yes	Yes	Yes	Yes	Yes
Constant	508.05*	537.54*	61.98	129.49	132.11*	43.61	36.30	117.37*
Observations	469	469	469	469	469	469	469	469
Number of hhid	286	286	286	286	286	286	286	286
Mean of Y	323.20	307.60	81.50	98.01	57.82	66.40	61.53	63.76

Note: Results were estimated using Radom-Effect model and using data collected from 2019 to 2021. Characteristics of household (income, household size, share of children and old people in the household, diversity of agricultural production) and household head (age, gender, marital status, education), and village dummies had been adjusted in the regression. Farmers referred to rural households who were still engaged in agricultural production. Number of hhid is the number of households net of duplicated observations. * refers to statistically significant at 5%.

Table S5 Short-run impact of COVID-19 on the consumption of 8 food categories for non-farmers

Variable	grains	vegetables	fruits	meat	eggs	aquacultu re	dairy products	legumes
COVID-19	-14.11	-17.01	5.26	-106.42*	15.75	53.38*	-30.61	58.90*
ln(income)	-21.25	-19.92	-1.61	1.67	-3.52	-7.95	13.60	-1.67
household size	-11.28	-29.34*	-13.28	1.00	-3.86	-11.41	-6.79	-9.41
old_share	9.65	46.87	-29.04	-39.40	-14.47	-95.16	-67.96	19.01
children_share	62.87	195.11	38.28	87.01	-0.47	-114.85	73.92	-11.50
Age	-0.41	-1.87	0.88	2.88	0.18	0.92	2.05	-0.28
Gender(male)	43.30	-99.12	-228.29	-49.43	8.18	-34.73	-44.56	-80.75
Marital status	-8.03	27.75	-61.70	8.55	-14.38	57.42	-27.53	-35.58
Education	-0.45	-4.94	3.39	4.65	-0.41	-0.97	2.47	0.91
Village control	Yes	Yes	Yes	Yes	Yes	Yes	Yes	Yes
Constant	374.21*	553.83*	218.73	-105.02	47.47	80.60	-16.25	89.66
Observations	129	129	129	129	129	129	129	129
Number of hhid	122	122	122	122	122	122	122	122
Mean of Y	319.90	296.40	66.37	84.91	53.76	62.02	61.51	60.85

Note: Results were estimated using Radom-Effect model and using data collected in 2019 and 2020. Characteristics of household (income, household size, share of children and old people in the household, diversity of agricultural production) and household head (age, gender, marital status, education), and village dummies had been adjusted in the regression. Non-farmers referred to rural households who did not produce any foods. Number of hhid is the number of households net of duplicated observations. * refers to statistically significant at 5%.

Table S6 Long-run impact of COVID-19 on the consumption of 8 food categories for non-farmers

Variable	grains	vegetables	fruits	meat	eggs	aquacultu re	dairy products	legumes
COVID-19	8.03	-24.61	6.54	-63.98	44.39	73.88*	-30.59	56.02*
ln(income)	-8.54	-10.37	11.52	33.40	1.14	-4.87	16.18	2.13
household size	-13.29	-34.34*	-16.90	-25.22	-3.97	-25.25*	-8.02	-12.54
old_share	57.00	39.04	-24.85	-132.81*	-40.97	-137.49*	-39.01	-34.43
children_share	165.83	87.68	115.96	260.98	-90.61	-65.55	117.63	-57.07
Age	-1.16	-0.80	1.59	6.29*	0.15	2.55	1.25	1.59
Gender(male)	17.98	-29.87	-224.11*	-96.73*	-13.20	-41.59	-30.03	-20.58
Marital status	-22.41	64.24	-25.38	88.14	4.97	88.57*	0.83	-32.75
Education	1.40	-4.45	5.31	4.98	1.19	-1.99	1.19	0.35
Village control	Yes	Yes	Yes	Yes	Yes	Yes	Yes	Yes
Constant	386.06*	443.24*	120.45	-301.75*	61.01	0.61	-5.29	-40.13
Observations	157	157	157	157	157	157	157	157
Number of hhid	142	142	142	142	142	142	142	142
Mean of Y	323.20	307.60	81.50	98.01	57.82	66.40	61.53	63.76

Note: Results were estimated using Radom-Effect model and using data collected from 2019 to 2021. Characteristics of household (income, household size, share of children and old people in the household, diversity of agricultural production) and household head (age, gender, marital status, education), and village dummies had been adjusted in the regression. Non-farmers referred to rural households who did not produce any foods. Number of hhid is the number of households net of duplicated observations. * refers to statistically significant at 5%.

Table S7 Impact of COVID-19 on dietary diversity and CFPS

Variable	Short-run		Long-run	
	dietary diversity	CFPS	dietary diversity	CFPS
COVID-19	-0.64*	0.24*	-0.75*	0.17
ln(income)	0.14	0.06	0.17*	0.06
household size	-0.09	-0.08	-0.03	-0.03
old_share	-0.34	-0.18	-0.32	-0.04
children_share	-0.02	-0.04	-0.53	-0.47
production diversity	-0.02	-0.01	0.03	0.00
Age	0.01	0.01	0.01	0.01
Gender(male)	-0.33	-0.24	-0.07	-0.14
Marital status	-0.18	0.41	-0.14	0.19
Education	0.05	0.01	0.06*	0.02
Village control	Yes	Yes	Yes	Yes
Constant	4.73*	1.16*	4.39*	1.23*
Observations	418	418	626	626
Number of hhid	325	325	345	345
Mean of Y	5.42	2.25	5.23	2.23

Note: Results were estimated using Radom-Effect model. Short-run impact was estimated using data collected in 2019 and 2020, and long-run impact was tested using data collected from 2019 to 2021. Dietary diversity was the number of food items consumed by each household. CFPS was the Chinese Food Pagoda Score. Characteristics of household (income, household size, share of children and old people in the household, diversity of agricultural production) and household head (age, gender, marital status, education), and village dummies had been adjusted in the regression. Number of hhid is the number of households net of duplicated observations. * refers to statistically significant at 5%.

Table S8 Impact of COVID-19 on dietary diversity and CFPS for farmers and non-farmers

Variable	Short-run				Long-run			
	Farmers		Non-farmers		Farmers		Non-farmers	
	dietary diversity	CFPS	dietary diversity	CFPS	dietary diversity	CFPS	dietary diversity	CFPS
COVID-19	-0.60*	0.30*	-0.58	0.38	-0.78*	0.17	-0.66	0.29
ln(income)	0.01	-0.03	0.30	0.14	0.19*	0.05	0.19	0.04
household size	-0.13	-0.10*	-0.01	-0.09	-0.05	-0.03	-0.02	-0.11
old_share	-0.29	-0.34	-0.43	-0.49	-0.05	0.07	-1.09*	-0.70*
children_share	0.12	-0.34	0.03	0.76	-0.38	-0.62	-0.03	0.73
production diversity	-0.06	-0.09*	---	---	0.06	-0.00	---	---
Age	0.02	0.02*	0.01	0.02	0.01	0.01	0.03	0.02
Gender(male)	-0.06	-0.21	-1.35*	-0.43	0.08	-0.07	-1.03	-0.46
Marital status	-0.22	0.44	-0.15	0.29	-0.21	0.06	-0.04	0.50
Education	0.03	-0.00	0.11	0.03	0.05	0.02	0.07	-0.00
Village control	Yes	Yes	Yes	Yes	Yes	Yes	Yes	Yes
Constant	5.07*	1.42*	4.72*	0.80	4.44*	1.44*	4.07*	1.10
Observations	289	289	129	129	469	469	157	157
Number of hhid	193	193	122	122	286	286	142	142
Mean of Y	5.42	2.25	5.42	2.25	5.23	2.23	5.23	2.23

Note: Results were estimated using Radom-Effect model. Short-run impact was estimated using data collected in 2019 and 2020, and long-run impact was tested using data collected from 2019 to 2021. Dietary diversity was the number of food items consumed by each household. CFPS was the Chinese Food Pagoda Score. Characteristics of household (income, household size, share of children and old people in the household, diversity of agricultural production) and household head (age, gender, marital status, education), and village dummies had been adjusted in the regression. Farmers referred to rural households who were still engaged in agricultural production, and non-farmers was the sample only includes rural households who did not produce any foods. Number of hhid is the number of households net of duplicated observations. * refers to statistically significant at 5%.

Table S9 Short-run impact of COVID-19 on the consumption of 8 food categories

Variable	grains	vegetables	fruits	meat	eggs	aquacultu re	dairy products	legumes
COVID-19	11.70	119.61*	19.70	12.75	10.65	29.66*	-8.67	28.04*
ln(income)	-1.01	11.48	5.78	12.26	0.81	-2.22	3.74	1.93
household size	-16.59*	-28.76*	-6.14	-6.34	-6.46*	-0.33	-8.33	-5.18
old_share	-51.73	15.67	-19.78	-28.24	-29.59*	-17.27	-19.80	36.37
children_share	-118.35	-31.29	-35.83	-10.36	-34.48	-127.63*	79.01	-16.83
production diversity	7.00	5.18	-1.67	-0.49	3.99	-1.11	1.06	-1.87
Age	0.41	-1.30	1.12*	1.03	0.09	-0.33	1.38*	-0.58
Gender(male)	31.30	-65.32	-39.03	1.08	10.96	-8.55	-44.47	27.13
Marital status	-34.10	53.63	-9.76	-13.54	-17.71	8.52	17.17	-55.36*
Education	-7.86*	-8.07*	2.82	0.33	-0.35	0.82	3.37*	-0.31
Village control	Yes	Yes	Yes	Yes	Yes	Yes	Yes	Yes
Constant	493.61*	573.82*	44.32	48.43	119.45*	94.33	-14.00	88.54*
Observations	418	418	418	418	418	418	418	418
R^2	0.15	0.30	0.07	0.09	0.09	0.14	0.09	0.16
Mean of Y	319.90	296.40	66.37	84.91	53.76	62.02	61.51	60.85

Note: Results were estimated using OLS model and using data collected in 2019 and 2020. Characteristics of household (income, household size, share of children and old people in the household, diversity of agricultural production) and household head (age, gender, marital status, education), and village dummies had been adjusted in the regression. * refers to statistically significant at 5%.

Table S10 Long-run impact of COVID-19 on the consumption of 8 food categories

Variable	grains	vegetables	fruits	meat	eggs	aquacultu re	dairy products	legumes
COVID-19	4.65	92.90*	37.98*	27.79*	14.07*	29.15*	-5.99	23.33*
ln(income)	8.74	11.35	23.72*	19.22*	11.86*	0.08	8.97	7.50
household size	-21.27*	-27.43*	-4.25	-12.77*	-5.09	-0.39	-7.96*	-3.46
old_share	0.68	17.22	15.12	-17.44	-25.19	-6.34	-1.66	26.99
children_share	51.04	-48.56	-10.02	61.95	-59.34	-108.16*	78.38	-41.34
production diversity	2.60	0.27	-0.93	1.71	1.88	-0.98	4.21	-2.86
Age	0.01	-0.44	0.53	0.45	0.05	0.06	0.15	-0.37
Gender(male)	23.60	-20.01	-35.49	-12.73	-1.72	13.41	-7.12	55.68*
Marital status	-4.32	15.66	-40.44	10.76	-6.93	-22.14	-14.30	-100.15*
Education	-7.78*	-6.72*	5.25*	1.38	-0.44	3.27*	3.33*	1.81
Village control	Yes	Yes	Yes	Yes	Yes	Yes	Yes	Yes
Constant	482.85*	495.35*	45.73	49.80	106.11*	23.27	26.07	78.61
Observations	626	626	626	626	626	626	626	626
R^2	0.11	0.22	0.10	0.07	0.07	0.11	0.09	0.12
Mean of Y	323.20	307.6	81.50	98.01	57.82	66.40	61.53	63.76

Note: Results were estimated using OLS model and using data collected from 2019 to 2021. Characteristics of household (income, household size, share of children and old people in the household, diversity of agricultural production) and household head (age, gender, marital status, education), and village dummies had been adjusted in the regression. * refers to statistically significant at 5%.

Table S11 Short-run impact of COVID-19 on the consumption of 8 food categories for farmers

Variable	grains	vegetables	fruits	meat	eggs	aquaculture	dairy products	legumes
COVID-19	9.45	137.28*	23.52*	28.23	8.15	28.08*	-7.23	25.35*
ln(income)	4.99	14.50	4.67	12.72	2.23	0.39	0.37	4.58
household size	-19.28*	-30.37*	-5.49	-10.58*	-8.56*	2.04	-8.91	-5.42
old_share	-75.79	-20.34	-25.91	-13.58	-38.80	6.76	7.24	57.53*
children_share	-170.12	-62.69	-49.92	-4.84	-41.43	-103.41	108.05	16.80
production diversity	10.81	3.43	-3.90	-3.99	5.06	1.34	0.56	0.40
Age	0.71	-0.78	0.77	0.15	-0.15	-0.90	1.12	-1.15*
Gender(male)	30.29	-68.66	2.65	33.89	16.48	-1.98	-39.96	61.87*
Marital status	-48.52	61.65	5.13	-46.18	-20.70	-19.94	33.02	-69.27*
Education	-8.61*	-10.25*	1.95	-0.60	-0.64	0.46	2.56	-0.90
Village control	Yes	Yes	Yes	Yes	Yes	Yes	Yes	Yes
Constant	529.93*	631.97*	54.68	157.61*	165.83*	108.71	-11.08	108.39*
Observations	289	289	289	289	289	289	289	289
R^2	0.19	0.36	0.13	0.17	0.12	0.12	0.10	0.21
Mean of Y	319.90	296.40	66.37	84.91	53.76	62.02	61.51	60.85

Note: Results were estimated using OLS model and using data collected in 2019 and 2020. Characteristics of household (income, household size, share of children and old people in the household, diversity of agricultural production) and household head (age, gender, marital status, education), and village dummies had been adjusted in the regression. Farmers referred to rural households who were still engaged in agricultural production. * refers to statistically significant at 5%.

Table S12 Long-run impact of COVID-19 on the consumption of 8 food categories for farmers

Variable	grains	vegetables	fruits	meat	eggs	aquaculture	dairy products	legumes
COVID-19	-1.24	100.59*	38.65*	27.39*	10.68	26.05*	-9.21	18.94*
ln(income)	12.49	13.83	26.91*	15.92*	15.98*	2.80	9.46	10.97*
household size	-24.98*	-27.05*	0.51	-9.38	-4.08	3.45	-8.58*	-2.09
old_share	-5.57	4.30	28.17	15.90	-16.08	29.91	22.87	48.48*
children_share	56.46	-57.02	-53.97	40.55	-63.78	-83.82	97.45	-18.18
production diversity	6.12	3.03	-3.90	-0.09	1.01	-0.36	6.82*	-3.64
Age	0.32	-0.42	-0.15	-0.55	-0.17	-0.73	-0.32	-0.97
Gender(male)	23.48	-22.34	-6.71	5.51	-1.86	25.64	1.58	75.50*
Marital status	-1.54	-0.41	-37.66	-11.35	-9.42	-57.21*	-23.89	-125.29*
Education	-9.68*	-7.53*	5.21*	-0.03	-1.03	3.47*	3.35*	1.78
Village control	Yes	Yes	Yes	Yes	Yes	Yes	Yes	Yes
Constant	499.71*	537.54*	61.59	129.49	125.71*	40.96	36.30	117.37*
Observations	469	469	469	469	469	469	469	469
R^2	0.13	0.23	0.13	0.08	0.08	0.11	0.10	0.15
Mean of Y	323.2	307.6	81.50	98.01	57.82	66.40	61.53	63.76

Note: Results were estimated using OLS model and using data collected from 2019 to 2021. Characteristics of household (income, household size, share of children and old people in the household, diversity of agricultural production) and household head (age, gender, marital status, education), and village dummies had been adjusted in the regression. Farmers referred to rural households who were still engaged in agricultural production. * refers to statistically significant at 5%.

Table S13 Short-run impact of COVID-19 on the consumption of 8 food categories for non-farmers

Variable	grains	vegetables	fruits	meat	eggs	aquaculture	dairy products	legumes
COVID-19	-1.18	-17.01	4.03	-49.14	16.08	45.13*	-12.39	51.01
ln(income)	-21.58	-19.92	-4.44	2.18	-3.45	-6.72	13.75	-0.91
household size	-9.68	-29.34*	-13.55	3.25	-3.97	-10.54	-8.85	-8.77
old_share	10.19	46.87	-32.45	-38.00	-14.21	-94.02	-74.71	20.91
children_share	53.70	195.11	37.16	36.50	1.61	-120.46	72.60	-10.83
Age	0.02	-1.87	1.03	2.42	0.20	1.34	2.07	-0.15
Gender(male)	50.62	-99.12	-228.62	-54.28	8.88	-27.66	-51.94	-74.31
Marital status	-12.33	27.75	-57.16	-2.87	-15.50	59.52	-19.14	-41.63
Education	-0.25	-4.94	3.39	5.18	-0.42	-0.82	2.53	0.90
Village Control	Yes	Yes	Yes	Yes	Yes	Yes	Yes	Yes
Constant	329.52	553.83*	211.65	-96.73	46.20	45.86	-17.56	80.53
Observations	129	129	129	129	129	129	129	129
R^2	0.25	0.36	0.27	0.22	0.16	0.34	0.19	0.23
Mean of Y	319.90	296.40	66.37	84.91	53.76	62.02	61.51	60.85

Note: Results were estimated using OLS model and using data collected in 2019 and 2020. Characteristics of household (income, household size, share of children and old people in the household, diversity of agricultural production) and household head (age, gender, marital status, education), and village dummies had been adjusted in the regression. Non-farmers referred to rural households who did not produce any foods. * refers to statistically significant at 5%.

Table S14 Long-run impact of COVID-19 on the consumption of 8 food categories for non-farmers

Variable	grains	vegetables	fruits	meat	eggs	aquaculture	dairy products	legumes
COVID-19	12.75	-2.20	18.07	-13.42	44.39	57.36*	-15.15	56.23*
ln(income)	-6.21	-10.04	10.27	32.03	1.14	-2.48	13.00	2.16
household size	-11.55	-34.03*	-21.65	-24.79	-3.97	-19.13	-10.54	-12.53
old_share	29.00	34.08	-41.52	-119.52*	-40.97	-139.83*	-67.34*	-35.15
children_share	113.67	73.17	133.66	250.89	-90.61	-107.67	78.55	-47.89
Age	-0.22	-0.63	2.05	5.59*	0.15	3.08	1.55	1.67
Gender(male)	24.62	-23.77	-228.79*	-107.30*	-13.20	-34.74	-35.31	-17.85
Marital status	-30.31	51.64	-10.42	87.02	4.97	83.79*	7.33	-36.99
Education	2.20	-4.28	4.78	5.57	1.19	-1.62	0.56	0.52
Village Control	Yes	Yes	Yes	Yes	Yes	Yes	Yes	Yes
Constant	332.48*	424.04*	99.45	-283.25*	61.01	-41.01	0.50	-47.93
Observations	157	157	157	157	157	157	157	157
R^2	0.22	0.33	0.25	0.25	0.17	0.36	0.18	0.19
Mean of Y	323.20	307.60	81.50	98.01	57.82	66.40	61.53	63.76

Note: Results were estimated using OLS model and using data collected from 2019 to 2021. Characteristics of household (income, household size, share of children and old people in the household, diversity of agricultural production) and household head (age, gender, marital status, education), and village dummies had been adjusted in the regression. Non-farmers referred to rural households who did not produce any foods. * refers to statistically significant at 5%.

Table S15 Impact of COVID-19 on dietary diversity and CFPS

Variable	Short-run			Long-run		
	dietary diversity		CFPS	dietary diversity		CFPS
	OLS	Poisson	OLS	OLS	Poisson	OLS
COVID-19	-0.63*	-0.63*	0.23*	-0.70*	-0.68*	0.17
ln(income)	0.13	0.13	0.05	0.18*	0.17*	0.06
household size	-0.09	-0.09	-0.08	-0.04	-0.03	-0.03
old_share	-0.37	-0.37	-0.19	-0.33	-0.33	-0.05
children_share	-0.02	-0.01	-0.07	-0.52	-0.51	-0.48
production diversity	-0.01	-0.01	-0.02	0.03	0.03	0.00
Age	0.02	0.02	0.01*	0.01	0.01	0.01
Gender(male)	-0.30	-0.28	-0.23	-0.04	-0.03	-0.13
Marital status	-0.17	-0.17	0.43	-0.15	-0.16	0.18
Education	0.05	0.05	0.01	0.06*	0.06*	0.02
Village Control	Yes	Yes	Yes	Yes	Yes	Yes
Constant	4.54*	---	1.11*	4.21*	---	1.21*
Observations	418	418	418	626	626	626
R^2	0.16	---	0.18	0.15	---	0.11
Mean of Y	5.42	---	2.25	5.232	---	2.232

Note: Results were estimated using OLS model and Poisson model. Short-run impact was estimated using data collected in 2019 and 2020, and long-run impact was tested using data collected from 2019 to 2021. Dietary diversity was the number of food items consumed by each household. CFPS was the Chinese Food Pagoda Score. Characteristics of household (income, household size, share of children and old people in the household, diversity of agricultural production) and household head (age, gender, marital status, education), and village dummies had been adjusted in the regression. * refers to statistically significant at 5%.

Table S16 Short-run impact of COVID-19 on dietary diversity and CFPS for farmers and non-farmers

Variable	Farmers			Non-farmers		
	dietary diversity		CFPS	dietary diversity		CFPS
	OLS	Poisson	OLS	OLS	Poisson	OLS
COVID-19	-0.56***	-0.57*	0.30**	-0.58	-0.54	0.41
ln(income)	0.01	0.02	-0.04	0.31	0.31	0.14
household size	-0.13	-0.14	-0.10*	-0.01	-0.01	-0.10
old_share	-0.35	-0.34	-0.35	-0.43	-0.43	-0.50
children_share	0.07	0.13	-0.36	0.04	0.01	0.76
production diversity	-0.05	-0.05	-0.09**	---	---	---
Age	0.02	0.02	0.02**	0.02	0.02	0.02
Gender(male)	-0.04	-0.02	-0.21	-1.33**	-1.35*	-0.43
Marital status	-0.23	-0.21	0.45	-0.10	-0.10	0.30
Education	0.03	0.03	0.00	0.11*	0.11*	0.03
Village Control	Yes	Yes	Yes	Yes	Yes	Yes
Constant	4.88***	---	1.40**	4.45***	---	0.74
Observations	289	289	289	129	129	129
R^2	0.17	---	0.26	0.24	---	0.27
Mean of Y	5.42	---	2.25	5.42	---	2.25

Note: Results were estimated using OLS model and Poisson model. Short-run impact was estimated using data collected in 2019 and 2020. Dietary diversity was the number of food items consumed by each household. CFPS was the Chinese Food Pagoda Score. Characteristics of household (income, household size, share of children and old people in the household, diversity of agricultural production) and household head (age, gender, marital status, education), and village dummies had been adjusted in the regression. Farmers referred to rural households who were still engaged in agricultural production, and non-farmers was the sample only includes rural households who did not produce any foods. * refers to statistically significant at 5%.

Table S17 Long-run impact of COVID-19 on dietary diversity and CFPS for farmers and non-farmers

Variable	Farmers			Non-farmers		
	dietary diversity		CFPS	dietary diversity		CFPS
	OLS	Poisson	OLS	OLS	Poisson	OLS
COVID-19	-0.75*	-0.75*	0.17	-0.63	-0.59	0.31
ln(income)	0.18*	0.17*	0.04	0.18	0.18	0.04
household size	-0.04	-0.04	-0.03	-0.04	-0.03	-0.12
old_share	-0.02	-0.02	0.07	-1.16*	-1.17*	-0.72*
children_share	-0.44	-0.45	-0.65	0.07	0.07	0.76
production diversity	0.05	0.05	-0.01	---	---	---
Age	0.01	0.01	0.01	0.03	0.03*	0.02
Gender(male)	0.10	0.12	-0.06	-1.03	-1.07*	-0.46
Marital status	-0.24	-0.24	0.06	0.04	0.02	0.50
Education	0.05*	0.05*	0.02	0.07	0.07	-0.00
Village Control	Yes	Yes	Yes	Yes	Yes	Yes
Constant	4.39*	---	1.43*	3.84*	---	1.05
Observations	469	469	469	157	157	157
R^2	0.16	---	0.15	0.24	---	0.20
Mean of Y	5.23	---	2.23	5.23	---	2.23

Note: Results were estimated using OLS model and Poisson model. Long-run impact was estimated using data collected from 2019 to 2021. Dietary diversity was the number of food items consumed by each household. CFPS was the Chinese Food Pagoda Score. Characteristics of household (income, household size, share of children and old people in the household, diversity of agricultural production) and household head (age, gender, marital status, education), and village dummies had been adjusted in the regression. Farmers referred to rural households who were still engaged in agricultural production, and non-farmers was the sample only includes rural households who did not produce any foods. * refers to statistically significant at 5%.
